# PHYSICAL CAPACITY IS ASSOCIATED WITH ATTENTION IN MIDDLE AGED ADULTS

**DOI:** 10.1093/geroni/igae098.4379

**Published:** 2024-12-31

**Authors:** Khalil Iktilat, Roy Tzemah-Shahar, Maayan Agmon

**Affiliations:** University of Haifa, Haifa, Hefa, Israel; University of Haifa, Haifa, Hefa, Israel; University of Haifa, Haifa, Hefa, Israel

## Abstract

Associations between physical capacity and various aspects of executive function, including attention, have been identified in older and younger cohorts, but the relationship among middle-aged individuals at the gateway of aging is largely understudied. The primary objective of this study was to investigate this relationship in middle-aged Muslims in Israel, a group experiencing chronic health disparities. This cross-sectional study included 255 participants (159 women), aged 51.29±4.26 (45-60), assessed using the six-minute walk test (6MWT) and 30-second sit-to-stand test (30STS) for physical capacity, and the Stroop Test and Trail Making Test (TMT) for selective and alternating attention, respectively. We found a significant positive correlation between the 30STS and Stroop-C scores even when controlling for age, sex, education level, and hemoglobin A1c, indicating that physical capacity is independently and positively associated with selective attention (β=0.132, P=0.057). In contrast, no correlation was found between the 30 STS and TMT (β=-0.107, P=0.107), suggesting that alternating attention is not associated with physical capacity. Interestingly, neither attention assessment was associated with the 6MWT, suggesting that among healthy, free living, middle-aged individuals, more challenging assessments should be used when examining associations between physical capacity and executive function. Our findings analyzed this past month, in time for late-breaking submission, highlight the nuances of the relationship between physical capacity and aspects of executive function middle aged adults. Interventions that include challenging physical tasks may reduce selective attention decline in middle-aged adults.

